# Rare acute generalized exanthematous pustulosis caused by iodixanol: A case report

**DOI:** 10.1097/MD.0000000000041301

**Published:** 2025-01-31

**Authors:** Xiuxiu Ma, Chaozhu He, Xiaohong Zeng, Xiaohua He, Yanfang Bi

**Affiliations:** a School of Nursing, Nanchang University, First Clinical Medical College, Jiangxi Medical College, Nanchang University, Jiangxi, China; b School of Nursing, Jiangxi Medical College, Nanchang University, Nanchang, Jiangxi, China.

**Keywords:** acute generalized exanthematous pustulosis, case report, iodixanol, mechanism, medication

## Abstract

**Rationale:**

Acute generalized exanthematous pustulosis (AGEP) is a rare delayed allergic reaction, which is mostly caused by drugs, but there are few reports of iodixanol in particular. At present, 4 cases induced by iodixanol have been reported in the literature, but none have been reported in China.

**Patient concerns:**

In this report, we report a case of AGEP induced by intravenous bolus infusion of iodixanol during multislice spiral CT enhanced scanning of the whole abdomen in a patient with rectal malignancy. A 35-year-old female patient presented with a larger rash/erythema on the head and neck and felt itchy.

**Diagnoses:**

A large erythema/rash and white, sterile pustules based on erythema spread from head, neck to the whole body in a short period of time. Blood cell results showed high levels of white blood cell, neutrophils, C-reactive protein and interleukin-10, and decreased levels of some immune indicators. Pathological biopsy results showed that there was immune cell infiltration around the superficial dermis.

**Interventions:**

Immediate avoidance of potential allergens and treatment with steroids, antihistamines and other medications, followed by adjustment with cyclosporine and human immunoglobulin.

**Outcomes:**

At 1 year’s follow-up, the patient did not develop any symptoms of discomfort and increased awareness of allergen prevention.

**Lessons:**

The reported case is an extremely rare and severe AGEP, typically characterized by pruritus, edema, large erythema/rash, dense white needle-like sterile pustules based on erythema/rash, and desquamation. After discontinuation of exposure to iodixanol, the patient received symptomatic supportive treatment, mainly drug response. She eventually recovered without recurrence. In the future, with the in-depth exploration of more studies, the use of Iodine Contrast Media will be more standardized and scientific.

## 1. Introduction

Acute generalized exanthematous pustulosis (AGEP) is a rare delayed adverse reaction with an incidence of only 1–5/1000,000 and was reported and named by Beylot et al^[1]^ in 1980. Clinically, it is characterized by the occurrence of patches of pinpoint, white, aseptic pustules on the basis of erythema and systemic symptoms such as fever, less organ involvement, most often accompanied by peripheral blood leucocytosis and neutrophilia, and may be accompanied by eosinophilia in approximately 1/3 of patients. According to the summary and analysis of the existing cases in relevant studies, approximately 90% of AGEP cases were caused by drugs, mainly antibiotics, and fewer reports were caused by Iodine Contrast Media, especially iodixanol.^[2]^ In addition, a small number of patients with AGEP may be due to family inheritance, viral infection, and exposure to poisons (such as mercury, spider exposure history, etc). The following is a rare case of AGEP caused by iodixanol in our hospital.

## 2. Case presentation

A 35-year-old female patient with rectal malignancy was admitted to the General Surgery Department of our hospital. To evaluate the primary lesion and distant metastasis, 80 ml of iodixanol (Production Batch No.: A2307212, Specification: 100:32 g[L]) was injected at a rate of 3.5 mL/second for multislice spiral CT enhanced scanning of the whole abdomen.

**Figure 1.**
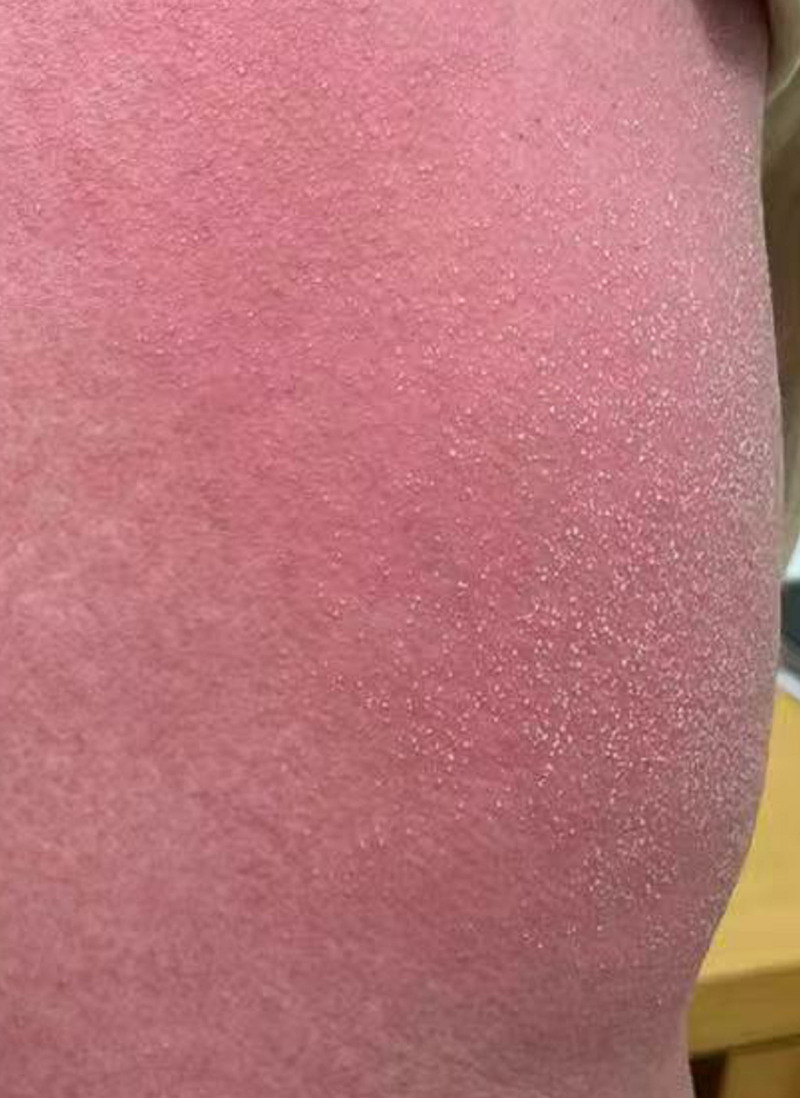
Large erythema/rash and dense, white, needle-like, sterile pustules based on erythema.

Within 12 hours, the patient began to itch and therefore scratch, which led to the redding of the skin, the development of erythema/rash on the neck of the jaw, the occipital area, and the upper part of her arms. The pressure faded and the mucosa was not damaged. We suspect it to be drug-induced dermatitis, and immediately avoid potential allergenic drugs. Moreover, ebastine (10 mg qd), feticasone propionate cream (external use) and diprospan (7 mg im) were given. The next day, her skin condition continued to worsen (pruritus redness and swelling aggravated; erythema/rash spread throughout the body, mainly concentrated in the trunk and roughly symmetrical; and the appearance of scattered or dense pinpoint-like size, white, sterile pustules based on erythema; Fig. 1), so a dermatological consultation was requested. Combined with the blood test results: white blood cell (11.22 × 10^9^/L), neutrophils (9.56 × 10^9^/L), C-reactive protein (11.68 mg/L), secrete interleukin (IL)-10 (37.75 pg/mL), and IL-22 (3.89 pg/mL) increased. Other laboratory parameters were unremarkable. Consultation diagnosis: AGEP? Dexamethasone sodium phosphate (10 mg ivgtt qd) combined with cetirizine hydrochloride tablets was given. In addition, intravenous drip of vitamin C (12 mL qd), calcium gluconate (10 mL qd), and sodium thiosulfate (640 mg qd). Meanwhile, wet compress of compound phellodendrin solution and strengthen hydration.

**Figure 2.**
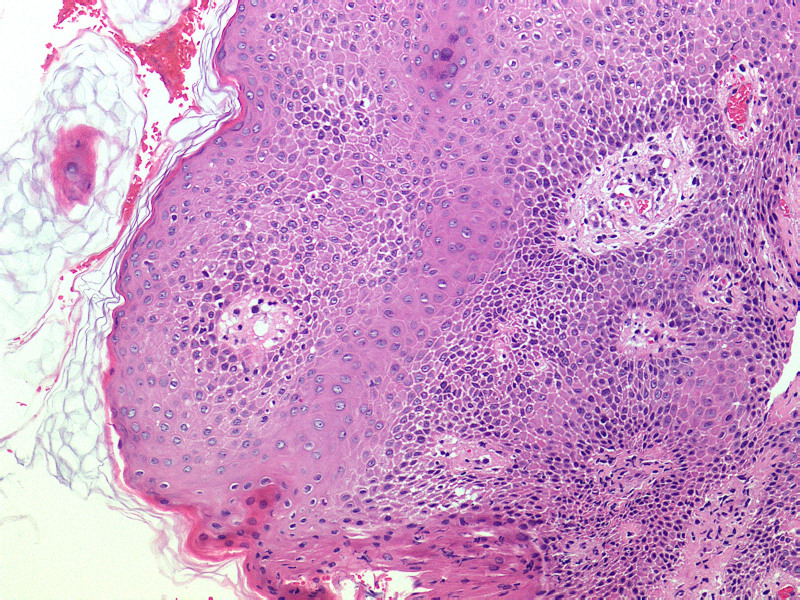
Spongy edema with superficial perivasculitis (hematoxylin and eosin staining, magnification ×100).

**Figure 3.**
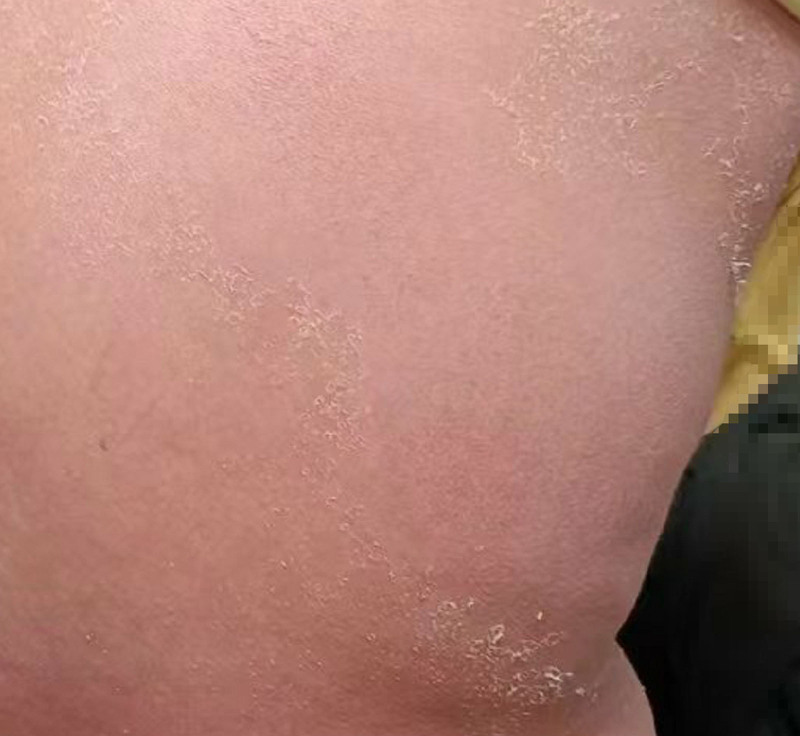
Desquamation, erythema lightening.

However, after 2 days of conventional treatment, the patient’s condition still worsened, so the patient was referred to the dermatology department for specialist treatment. On the day of transfer, the lesion on right thigh was biopsied for differential diagnosis. Biopsy results: epidermal basket reticular keratosis, acanthosis with mild spongy edema, focal scattering and few neutrophils. Superficial dermal perivascular lymphocyte infiltration with scattered intermediate centriocytes and individual eosinophilic infiltration were observed (Fig. 2). This finding supports our suspicions about AGEP. In view of the decreasing trend in immune function (immune indicators showed varying degrees of decline, such as CD4+ T cells, CD8+ T cells, and granzyme/perforin, etc), cyclosporine(125 mg q12h) and human immunoglobulin were added to regulate immune function, while recombinant human epidermal growth factor was used for external use. On the second day after transfer, the patient’s local skin began to desquamate, and her condition gradually improved (Fig. 3). On the 6th day after transfer, the skin erythema was light in color and there was light yellow pigmentation at the edge of the erythema, and many papules were scattered in both lower limbs. The patient was referred to the general surgery department and was instructed to continue using human immunoglobulin, the amount of cyclosporin used was reduced by half (3 tablets in the morning and 3 tablets in the evening), and epasteine was applied externally when pruritus occurred. After 3 days, the skin condition completely recovered.

The patient was followed up by telephone twice within 1 year of discharge, during which time no abnormalities were observed.

## 3. Discussion

As a nonionic isotonic dimer, AGEP induced by iodixanol has a very low incidence and has rarely been reported. This is the first reported case of AGEP induced by ioxanol in China. The internal mechanisms have not been clarified, and some changes in immune-related indexes still need to be explored (e.g., IL-10 is elevated in Stevens Johnson Syndrome/Toxic Epithelial Neoplasia [SJS/TEN] but has not been confirmed in AGEP^[3]^), and most of them are thought to be T-cell-mediated delayed hypersensitivity reactions.^[4]^ At present, it is mainly timely identification and symptomatic treatment, and the differential diagnosis and drug therapy are discussed below.

Differentiating AGEP from other adverse drug reactions, such as drug rash with eosinophilia and systemic symptoms (DRESS) and Stevens-Johnson syndrome/toxic epidermal necrolysis and loosening (SJS/TEN), is challenging. Mucosal involvement was more prevalent in DRESS (50%) and SJS/TEN (>90%) than in AGEP, and DRESS often involves organs, whereas AGEP rarely involves either mucosa (20%) or organs.^[5,6]^ In terms of pathogenesis, DRESS and SJS/TED have longer incubation periods (4~8 weeks and 4~28 days, respectively) and duration of illness, and their conditions can recur despite discontinuation of the relevant drugs.^[6,7]^ In addition, compared to the mild eosinophilia that has been reported in about one third of AGEP cases, significant eosinophilia was more common in patients white DRESS (>50% of cases) and not as high in patients whit SJS/TED.^[5,7]^ In a further addition to this, tissue biopsy is also helpful for differential diagnosis (DRESS: epidermal and dermal vacuoles, eosinophilic infiltration; SJS/TED: whole-layer keratinocyte necrosis). AGEP was considered in this case. The fact that <12 hours elapsed between drug onset and skin reaction is consistent with the described incubation period of 48 hours.^[8]^ In addition, fever, rash/pustules manifestations, increased white blood cell, duration of illness, and histopathological changes are other arguments in favor of AGEP. A score of 11 according to the European Study of Severe Cutaneous Adverse Reactions scoring criteria^[2]^ means that a definite diagnosis of AGEP can be made. After the onset of the disease, the patient did not use other than iodixanol. In combination with the patient’s and her family’s denial of skin history including psoriasis, a history of viral infection, a history of toxic exposure, and the existence of alcohol allergy, we investigated the patients who received the same batch of iodixanol during the same period and did not have any adverse reactions. Therefore, we highly suspect that this case was caused by iodixanol and ruled out product quality issues.

In terms of medication, the World Allergy Organisation and the European Academy of Allergy and Clinical Immunology published relevant guidelines in 2020 and 2014, respectively, recommending antihistamines as second- and third-line agents for the treatment of allergic reactions. The European Academy of Allergy and Clinical Immunology also noted that the combination of H1 and H2 antihistamines may be more beneficial than H1 antihistamines alone.^[9]^ In this paper, the combination of ebastine (H2 antihistamine) and cetirizine (H1 antihistamine) was recommended in accordance with guidelines. However, a study evaluating the efficacy of cimetidine (H2 antihistamine) and diphenhydramine (H1 antihistamine) alone and in combination for the treatment of acute anaphylaxis showed no additional benefit.^[10]^ In this regard, the economic benefits of the combined application of antihistamines in this study remain to be studied. The 4 reported cases of iodixanol-induced AGEP reported thus far were cured by discontinuing the sensitizing drug along with corticosteroids and/or steroids.^[11–13]^ The difference is that the patient did not experience significant remission through conventional measures, but she experienced improvements after immunomodulation with cyclosporine and human immunoglobulin. A retrospective study by Creadore et al^[14]^ found that 2.9% (10/340) of AGEP patients were improved or completely cured after using cyclosporine. Lee et al^[15]^ reported a case of AGEP treated with cyclosporine when topical steroids failed to control the development of the rash. In addition, regarding the recombinant human epidermal growth factor we used, Song et al^[4]^ used it in AGEP patients to promote wound healing.

## 4. Conclusion

This paper focuses on the differential diagnosis of AGEP with respect to various findings such as the incubation period, involvement, symptomatic manifestations, histopathology, etc, and analyses the therapeutic effects of each drug. It emphasizes the possibility of iodixanol causing serious adverse skin reactions and the need for in-depth study of AGEP-related influencing factors and occurrence mechanisms. In the future, closer clinical management and more accurate diagnosis are still needed to identify and discontinue the triggering drugs in time, standardize the management of the disease, and provide more effective therapeutic benefits to patients.

## Acknowledgments

We would like to express our gratitude to the patient for granting permission to use their clinical data in this paper and for the publication of this research.

## Author contributions

**Formal analysis:** Xiuxiu Ma, Xiaohua He.

**Investigation:** Xiuxiu Ma, Yanfang Bi.

**Resources:** Xiaohong Zeng.

**Supervision:** Chaozhu He, Xiaohong Zeng.

**Writing – original draft:** Xiuxiu Ma.

**Writing – review & editing:** Chaozhu He, Xiaohong Zeng.
